# Critical View on the Importance of Host Defense Strategies on Virus Distribution of Bee Viruses: What Can We Learn from SARS-CoV-2 Variants?

**DOI:** 10.3390/v14030503

**Published:** 2022-02-28

**Authors:** Niels Piot, Guy Smagghe

**Affiliations:** Laboratory of Agrozoology, Department of Plants and Crops, Faculty of Bioscience Engineering, Ghent University, Coupure Links 653, 9000 Ghent, Belgium

**Keywords:** virus tolerance, virus resistance, host, distribution, bee virus variants

## Abstract

Bees, both wild and domesticated ones, are hosts to a plethora of viruses, with most of them infecting a wide range of bee species and genera. Although viral discovery and research on bee viruses date back over 50 years, the last decade is marked by a surge of new studies, new virus discoveries, and reports on viral transmission in and between bee species. This steep increase in research on bee viruses was mainly initiated by the global reports on honeybee colony losses and the worldwide wild bee decline, where viruses are regarded as one of the main drivers. While the knowledge gained on bee viruses has significantly progressed in a short amount of time, we believe that integration of host defense strategies and their effect on viral dynamics in the multi-host viral landscape are important aspects that are currently still missing. With the large epidemiological dataset generated over the last two years on the SARS-CoV-2 pandemic, the role of these defense mechanisms in shaping viral dynamics has become eminent. Integration of these dynamics in a multi-host system would not only greatly aid the understanding of viral dynamics as a driver of wild bee decline, but we believe bee pollinators and their viruses provide an ideal system to study the multi-host viruses and their epidemiology.

## 1. Viral Defense Strategies of the Host and Their Implications on Viral Dynamics

While viral infections may sometimes wreak havoc on host populations, often triggered by a host switch, they are indispensable components of a well-functioning ecosystem [1,2]. Here, hosts and viruses have co-evolved and live in a dynamic equilibrium, where viruses act as a top-down force controlling host populations. While viruses have evolved a diverse range of host infection routes, the host’s defenses can generally be categorized into two main defense strategies to combat viral infections. Either the hosts can prevent, eliminate, or significantly reduce a viral infection which is generally referred to as viral resistance. A second defense strategy is called viral tolerance. Here, the host can ‘tolerate’ the viral infection and replication without any major detrimental effects on its health [3].

Viral resistance has been investigated for a long time in animal studies, while viral tolerance has only seeped in during the last decade mainly fueled by research from plant pathogens, where tolerance research has a longer history. Both defense strategies benefit the host’s fitness but have different outcomes for viral fitness. Resistant hosts generally reduce the viral prevalence in the population, whereas tolerant hosts mostly have a positive impact on viral prevalence [4,5]. The latter can be induced by so called super-spreaders. These super-spreaders are tolerant hosts, experiencing no or only mild health effects of an infection, and they infect a disproportional number of new hosts, far exceeding the general reproduction number of the virus or pathogen at hand. Here, one could refer to the classic textbook example of Mary Mallon, dubbed “Typhoid Mary”, an asymptomatic carrier of *Salmonella enterica serovar typhi*, who infected multiple people. Yet, one does not need to go that far in history for such examples as the current SARS-CoV-2 pandemic is marked by several super-spreader events, where people with no or very mild symptoms infect a disproportional amount of people [6,7].

While these examples are all in a single host species, *Homo sapiens* (although both SARS-CoV-2 and *S. enterica* can infect multiple host species [8,9]), super-spreading can also occur between host species as most viruses readily circulate between host species, which often hampers virus eradication [10]. Differences in defense mechanisms between host species may result in one species coping better with infections than others and/or transmitting a disproportional amount of pathogens [11]. For bee viruses, studies on viral defense strategies on individual level scarce are mostly investigated in conjunction with other factors, such as nutrition or pesticide exposure, which complicates the interpretation of ‘pure’ viral tolerance or resistance [12,13,14]. There are ample prevalence studies that report links with the viral prevalence in wild bees and the presence of honeybees [15,16,17,18]. It should, however, be noted that transmission directionality cannot be inferred from these studies, which are correlational.

Yet, if one would assume that managed honeybee hives are indeed a source of the virus spilling over to wild bees, it remains to be answered if this is due to the defense strategies on the individual species level (e.g., they are more tolerant to certain viruses compared to wild bees), if this is just a numbers game (where the social honeybee with thousands of individuals per hive massively spreads the virus to the community, and individual deaths do not impede colony development), if *Varroa destructor* is the driving force, or a combination of these. *V. destructor*, an ectoparasitic mite of honeybees, has indeed shown to play a major role in the viral dynamics in honeybees, where it can effectively vector several viruses and increase viral load in parasitized colonies [19,20,21]. Here, Varroa-infested honeybee hives, harboring very high loads of virus may form a super-spreader to the environment, increasing viral exposure to other bee species [22]. Furthermore, the transportation of honeybee hives may also render these hives super-spreaders, transporting viruses across large distances and different ‘local’ bee communities.

Interestingly, it was recently shown that naturally, Varroa-resistant honeybee populations are more tolerant to viruses transmitted by this vector, such as *Deformed wing virus* (DWV) and *Acute bee paralysis virus* (ABPV) as these mite-resistant populations had reduced mortality, yet showed a similar infection dynamic compared to mite-susceptible honeybee populations [14]. The relation with *V. destructor,* which has a nearly worldwide distribution [19], and viral dynamics in honeybees has complicated the study of host defense strategies in honeybees (*Apis mellifera*), the most studied bee species in the bee community. Studies on species differences in viral defense strategies of bees are currently still lacking, yet we believe this would greatly benefit our understanding of multi-host viral dynamics, as we elaborate below.

## 2. Bee Pollinators as Study System for Multi-Host Viral Dynamics

The viral epidemiology in bee pollinators greatly lends itself as a study system to address the role of tolerance and resistance in different host species in a multi-host viral landscape, as bee pollinators share a specific ecological niche, namely flowers. Flowers provide the main source of food for bees. Visitations of these flowers hence mediate an indirect contact between individuals and species, which enables viral transmission via fecal contamination of the flowers, as most of the viruses that infect bees have an oral-fecal transmission route [21,22,23,24].

Here, the plant-pollinator network provides an ideal system to trace viral spread and encounter locations. Recently, several studies have identified the pivotal role of plant-pollinator networks in pollinator disease dynamics [25,26,27]. One could draw the parallel with super-spreading events documented for SARS-CoV-2 with the visitation or aggregation of multiple bees on the same flower, or the frequent visitation of flowers by multiple bees, for example, due to shortage of sufficient flowers [25,28,29]. Although flowers provide the main route of inter-species transmission in bees, intra-species transmission, especially in social bees (e.g., *Apis* sp. and *Bombus* sp.), mainly occurs in their colonies where high numbers of individuals live closely together. Additionally, as mentioned before, for *Apis* spp. the vector *V. destructor* also plays a major role in viral transmission for several bee viruses [19,20,21].

While research on the role of flowers on transmission is beginning to take ground, research on viral defense strategies of the hosts, i.e., resistance and tolerance, and its role in viral dynamics is still mostly lacking. Yet, bee research would greatly benefit from such studies to better understand what drives current emerging infectious disease in both wild and managed bees, as disease is one of the main drivers of the current bee decline [30]. Ever since the initial virus discoveries in honeybees, mostly by B. Bailey, B. Ball and colleagues, now more than 50 years ago, viral research has progressed a lot in honeybees, as well as in wild bees [31]. Sparked by the global decline of wild pollinators and colony losses of managed honeybees, research on viruses in both wild and managed bees has exploded over the last decade. Knowledge on the interaction with the bee host has been gathered for several viruses, where honeybees and to a lesser extent bumblebees are the main investigated host species.

Multiple studies have been undertaken to identify immune pathways (which are remarkably similar between bee species [32]) and gene regulation involved in the antiviral response of these bee hosts. Apart from the well-studied antiviral RNA interference (RNAi) response, other immune responses have been identified to be involved in antiviral responses in both honeybees and bumblebees. These include the Jak/Stat, Imd and other pathways, as well as the Toll pathway. In the latter, the gene family NF-κB appears to play a key role in immunosuppression, which can result in the destabilization of virus infection for cover to overt infection [20,33]. As further research on these bee host-virus interactions continues, our understanding of these mechanisms keeps growing [34,35,36]. This knowledge can be of great assistance in understanding the immunological background of what makes a host tolerant to a certain virus, as data from current research in non-bee hosts suggests there appears to be a lack or suppression of the immune response to the infection [37]. However, although the immune pathways of bees are relatively well characterized, knowledge on viral cell entry of bee viruses is a crucial factor that is currently missing to obtain a full picture of host viral dynamics. Unlike SARS-CoV-2, where the interaction with the ACE2 receptor is well described to induce host cell entry [38], no such data is currently available for bee viruses. This is in part due to the lack of bee cell lines, which greatly aid the ease of studying this process [39]. Viral cell entry of both *Dicistoriviridae* and *Iflaviridae* (both Picornavirales) is assumed to be via receptor-mediated endocytosis, but the precise cellular receptor(s) of these viruses are currently not known [40].

Data of the genomes of several bee species can further aid research on the bee’s viral immune response, as well as data from multiple transcriptomic studies of bees under viral infection and analogies with arthropods, such as the model insect *Drosophila melanogaster* and its interactions with viruses. Furthermore, genome information of the bee hosts has greatly assisted the discovery of undescribed viruses using metagenomics, as they simplify the subtraction of bee sequences.

An interesting study, where genome information was of assistance, reports on the integration of a part of the viral genome of Israeli acute paralysis virus (IAPV) in the honeybee (*Apis mellifera*) genome [41]. The study continues to show the detection of IAPV-derived transcripts and finds that bees harboring the viral segment in their genome, survive injection with IAPV, contrary to those that do not harbor the viral segment [41]. Although the study does not provide much information on the precise origin of the used honeybees, the fact that they do not find the endogenous viral element (EVE) of IAPV in all tested bees, and the fact that this phenomenon has not been reported elsewhere, strongly suggests that this is an unfixed EVE (i.e., the EVE is not fixated in the host genome and not passed on through generations) in the tested gene pool. Although there is currently no clear understanding of the precise mechanisms of integration, endogenous viral elements of non-retroviruses (nrEVEs) are found in a wide range of taxa. nrEVEs are quite abundant in mosquitoes where they are often studied and likely involved in antiviral immunity by producing Piwi-interacting RNAs ([piRNAs] small non-coding RNA fragments which are involved in a wide range of functions, including the regulation of gene expression and fighting viral infections) [42,43,44,45,46]. This has recently been shown in *Aedes aegypti*, where nrEVE with very high sequence similarity to cell-fusing agent virus (CFAV) were found in the mosquito genome [42]. When they removed the nrEVE (using CRISPR-Cas9), viral replication was increased in the ovaries alongside the reduction of CFAV-derived Piwi-interacting RNAs [42].

## 3. Viruses and Their Variants

The majority of currently described bee viruses are RNA viruses (*Piornavirales*) with a positive single stranded RNA (ssRNA) genome, just like SARS-CoV-2 (*Nidovirales*) (see Table 1). Whether the currently described bee virus diversity represents the true virological biodiversity of bee viruses is not known since the bulk of ‘newly’ described bee viruses has been performed by high throughput transcriptomic studies. This method favors the detection of these RNA viruses, particularly those containing a poly-A tail. Nonetheless, most currently described viruses, with clear pathology in bees, all have a positive ssRNA genome [23]. The mutation rate of RNA viruses (~10^−6^ to 10^−4^ substitutions per site per cell infection [s/n/c]) is generally higher than that of DNA viruses (~10^−8^ to 10^−6^ s/n/c), due to lack of proofreading activity of RNA viruses [47]. Hence, many RNA viruses are genetically heterogeneous and exist as a quasispecies. These quasispecies can either live as a cloud of mutants around a certain master variant, or several master variants can be present, each having its own cloud of mutants. However, not all RNA viruses have this very high mutation rate. Viruses of the *Coronaviridae* family (which have one of the largest genomes of RNA viruses, and includes SARS-CoV-2) possess a highly conserved exonuclease (nsp14) with proofreading function [48]. This likely explains the lower mutation rate of coronaviruses (4 × 10^−6^ s/n/c) compared to other RNA viruses [49]. Interestingly, recent research has shown that recurrent deletions (which cannot be corrected by a proofreading enzyme) in the SARS-CoV-2 genome (more specifically the spike glycoprotein) likely accelerate viral antigenic evolution, circumventing the ‘limiting’ effect of the proofreading enzyme [50].

While the bulk of data on SARS-CoV-2 variants originates from population/community data, some studies have shown within (human) host viral evolution [53]. However, most of the intra-host variants were not observed as polymorphic in the population data which suggests a strong bottleneck during transmission [53,54]. As it is estimated that around 10 to 1000 virions are needed to induce a new infection, less dominant variants are less likely to pass the transmission bottleneck [55].

This is in sharp contrast with the current data on the number of virions needed to induce oral infection with several bee viruses in honeybees and bumblebees which is several orders of magnitude larger (10^6^ to 10^10^, depending on the virus and bee host) [56,57,58,59,60]. Sequence information on the viral genomes of most bee viruses has enabled research on the viral adaptation of bee viruses, which also gained a lot of attention lately. Here, most studies worked with the DWV-complex, a quasispecies for which three master variants are identified (DWV-A, DWV-B and DWV-C), based upon nucleotide identity and differences in effects on replication, virulence, and pathology [61,62,63,64,65]. Furthermore, despite mutations, recombination events between variants have also been identified [60,66,67] and are known to play a role in the generation of new variants with picorna-like viruses [68]. DWV is predominantly found in honeybees (although it can infect other bee hosts as well [21,69]). This virus can be regarded as a special case in the bee viruses as its epidemiology and adaptation is mainly controlled by the presence of its vector, *V. destructor* (only present on honeybees) [19,22,66]. Not only does the presence of *V. destructor* increase viral titer in the honeybee hosts, but it also strongly decreases viral strain diversity and likely functions as a bottleneck for viral diversity [67,70]. Here, only a very small inoculum is needed to induce infection through injections (as performed by *V. destructor* parasitizing honeybees) [71], which is in the same order of magnitude as for SARS-CoV-2. Recently, a fourth major variant has been identified (DWV-D) via a sequencing analysis of old honeybee samples [72]. However, a large effort to detect the current presence of this variant, by screening RNA libraries from different bee hosts and varroa mites, across the world, did not result in the detection of the DWV-D variant. Either this variant has gone extinct, is replaced by or pushed back by other variants, or is only present in a very restricted geographical area [72]. The change in dominant variants of DWV is also documented in the UK and USA, where over the last 5 to 10 years the previously dominant DWV-A variant is being replaced by the DWV-B variant [73,74,75,76,77]. The underlying nature of this variant shift has not been fully clarified. Yet some studies point to the higher replication and reduced virulence costs of DWV-B infection, which may be due to different tissue tropism [64,73,78,79]. The virus may evolve to less severe and more contagious/transmissible variants, causing less harm to the host. However, other studies find that DWV-B is more virulent compared to DWV-A [80,81]. The discrepancies between studies may be attributed to the complex host-genotype-virus interaction determining the virulence of a certain variant [64], or other confounding factors, such as differences in the presence of *V. destructor* between studies. Further research is needed to elucidate this. Variant shifts have also been observed during the SARS-CoV-2 pandemic, and currently the Omicron SARS-CoV-2 (B.1.1.529) variant has replaced the more severe and less transmissible Delta SARS-CoV-2 (B.1.617.2) variant [82]. Here, several studies have found that the Omicron variant has a lower hospitalization rate as well as decreased risks of severe clinical symptoms compared to the Delta variant [83,84,85,86]. However, due to the higher transmissibility of the Omicron variant, i.e., reaching and infecting more people, some countries report a net increase in absolute numbers of hospitalization compared to the Delta variant, yet still associated with a lower clinical severity [87]. Theoretically, a virus is expected to evolve towards an evolutionary stable virulence, which is an optimum found in the trade-off between transmission and the negative effects of a viral infection, which may hamper its transmission (e.g., mortality) [88]. However, vaccinations (i.e., those that alleviate the host’s symptoms and do not prevent transmission, e.g., BNT162b2 vaccination against SARS-CoV-2 [89]) may alter the ‘natural’ evolution of viruses as it diminishes the negative effects of a virus in vaccinated infected hosts, which may favor the evolution towards higher virulence in unvaccinated hosts [90]. Currently, SARS-CoV-2 appears to be evolving towards higher transmission and lower virulence.

Unlike, SARS-CoV-2 there are currently no commercially available vaccines or treatments for bee viruses. Although there have been initiatives with the use of dsRNA to reduce the viral titer by triggering the RNAi immune response [36,91,92,93], products are not commercially available [94]. Viral evolution is hence, not ‘disturbed’ by vaccination in bees. However, as *V. destructor* plays a major role in honeybee viral dynamics, beekeeping practices, such as treatments against *V. destructor* may affect viral evolution. One of the suggested mechanisms underlying the shift of DWV-A towards DWV-B is that DWV-B may be better adapted to vector-mediated transmission via *V. destructor* [78,95,96]. Furthermore, Norton et al. [96] showed that the prevalence of DWV-A is highly dependent on the mite presence and density. DWV-B, on the other hand, was far less dependent on mite presence and always had a higher prevalence and infection load in both low and high mite densities compared to DWV-A [96]. As good beekeeping practices include treatment against *V. destructor* mites (which, when applied appropriately can significantly reduce mite levels, yet it does not fully eradicate them [97]), this practice may also contribute to the shift towards a dominant DWV-B. However, further research is still needed to elucidate precise mechanisms underlying this variant transition of DWV in bees.

Contrary to humans, bees have only a limited traveling range, and the global dissemination of certain bee virus variants is mostly driven by human transportation and trade of honeybees, or their products [21,98]. Therefore, the speed and scale of variant dissemination are much lower as compared to the SARS-CoV-2 pandemic. Although the high population density of honeybee hives, especially in large apiaries, provides the ideal ‘breeding ground’ for new variants to emerge, their dissemination will be slower compared to SARS-CoV-2. The unprecedented magnitude of viral (genome) sequencing on a global scale of SARS-CoV-2 will provide valuable data on viral evolution in general and variant dissemination, which can be used to better understand variant shifts in bee viruses as well as bee virology and epidemiology in general.

## 4. Conclusions

Research on bee viruses has come a long way, rapidly progressing over the last few years. It is our opinion that with the current knowledge the time is right to integrate and look at the host’s defense strategies and their role in viral dynamics in a multi-host system. SARS-CoV-2 and the few studies on animals highlight the importance of these defense strategies in shaping viral epidemiology, such as virus tolerance, which can result in super-spreaders [6,37].

## Figures and Tables

**Table 1:** Comparison between SARS-CoV-2 and two common bee virus families.

Genome	Order	Family	Virus	Genome Size ^£^	Virion Size	Enveloped	Hosts	Mutation Rate	Infection Dose
(+)ssRNA	*Nidovirales*	*Coronaviridae*	SARS-CoV-2	~29–30 kb	~120 nm	yes	Vertebrates (incl. humans, bats)	~4 × 10^−6^ s/n/c	10–1000
(+)ssRNA	Picornavirales *	*Dicistroviridae*	16 described bee-infecting viruses (e.g., Aki-virus complex)	~8.5–10.2 Kb	~30 nm	no	Invertebrates (incl. *Apis* spp., *Bombus* spp.)	~10^−6^ to 10^−4^ s/n/c	10^6^ to 10^10^ (oral)
		*Iflaviridae*	12 described bee-infecting viruses (e.g., DWV-complex)	~8.8–9.7 kb					10^2^ to 10^3^ (injection) ^$^

* In this table we have limited ourselves to the *Picornavirales* and here report on two virus families within this order found in bee species, which currently contain most currently described bee viruses. For an extensive list of currently described viruses found in bees we refer to [23]. ^$^ Injection studies have been performed in both *Apis* spp. and *Bombus* spp. yet only *Apis* spp. are parasitized by *V. destructor*, a vector of several bee viruses, which punctures the bee body; reported injection doses are based upon injection studies. ^£^ Genome size estimates: SARS-CoV-2: [51]; *Dicistroviridae* and *Iflaviridae*: [52].
